# Direct Solid-State Polymerization of Highly Aliphatic PA 1212 Salt: Critical Parameters and Reaction Mechanism Investigation Under Different Reactor Designs

**DOI:** 10.3390/polym18010101

**Published:** 2025-12-29

**Authors:** Angeliki D. Mytara, Athanasios D. Porfyris, Constantine D. Papaspyrides

**Affiliations:** 1 Laboratory of Polymer Technology, School of Chemical Engineering, National Technical University of Athens, Zographou Campus, 157 80 Athens, Greece; amytara@mail.ntua.gr (A.D.M.); 2 Research Lab of Advanced, Composite, Nano-Materials and Nanotechnology (R-NanoLab), School of Chemical Engineering, National Technical University of Athens, 9 Heroon Polytechniou, 157 73 Athens, Greece; adporfyris@mail.ntua.gr (A.D.P.)

**Keywords:** long-chain aliphatic polyamides, direct solid-state polymerization, polyamide synthesis, solid-melt transition (SMT), FTIR-ATR

## Abstract

The present work aims to synthesize polyamide 1212 (PA 1212) via direct solid-state polymerization (DSSP), starting from its solid salt precursor. The DSSP of aliphatic polyamide salts has been found to proceed through melt intermediates, in harmony with the well-documented solid-melt transition (SMT) mechanism. However, PA 1212 salt is anticipated to deviate from this model due to its strongly hydrophobic nature. The reaction was initially investigated at the microscale in a thermo-gravimetric analysis (TGA) chamber and then scaled up to the laboratory scale. The influence of reactor design, reaction temperature, and residence time was examined. DSSP products were characterized in terms of molecular size and morphological properties. At the same time, a novel protocol was developed for qualitatively monitoring the progress of polymerization via Fourier Transform Infrared Spectroscopy-Attenuated Total Refraction (FTIR-ATR) analysis. Emphasis was given on the resulting morphology examined via Scanning Electron Microscopy (SEM imaging). Although DSSP has been found to proceed through a quasi-SMT, significant differences are observed compared to the classical mechanism established in the literature. This paper reveals that the limited surface softening or agglomeration phenomena encountered are mostly associated with the hydrophobic structure of the PA 1212 salt.

## 1. Introduction

Long-chain aliphatic polyamides (PAs) bridge the gap between conventional polyamides and polyethylene (PE), as they combine both the high melting point and mechanical strength of polyamides with higher melt flow yield and resistance to water and polar solvents [1,2,3,4]. In particular, polyamides such as PA 612, PA 1012, and PA 1212 have a lower density of polar amide groups in their structure; therefore, they outmatch PA 66 and PA 6 in terms of water absorbance and flexibility. Thus, they are more suited to applications where water exposure and maintenance of mechanical properties are necessary (e.g., fishing nets) [1]. Moreover, current developments in the synthesis of polyamide monomers, such as sebacic and dodecanedioic acid via renewable resources, present new potential for the synthesis of partially or even fully biobased polyamides, thus raising a sustainability aspect [5,6,7,8,9,10,11,12,13]. PA 1212 is an established and commercially available long aliphatic chain polyamide that offers especially low hygroscopicity and thermal and dimensional stability and can be processed at a range of temperatures. PA 1212 can replace PA 12 in some applications (such as 3D-printing and selective laser sintering) as an equally effective and potentially “greener” alternative (due to the 1,12-dodecanedioic acid used, being one of the most known bio-sourced acids) [5,6,7,10,11]. PA 1212 is also being studied as a matrix for the development of advanced materials, such as thermoplastic elastomers, composites with polyurethane, polystyrene, and carbon nanofibers, to increase toughness, rigidity, and compression resistance [14,15,16,17,18,19,20].

The patent and open literature on the synthesis of PA 1212 include typical melt polymerization techniques. In most sources, an initial pressurized solution-melt step is further followed by venting to remove the polycondensation water. Then, a second melt polymerization step follows at a higher temperature, either by using purge gas flow or vacuum [14,18,21,22,23]. In contrast, in this work, the synthesis of PA 1212 via DSSP is investigated. DSSP involves heating the solid reacting mass, i.e., the polyamide salt, under an inert gas flow, at a temperature lower than both its melting point the melting point of the corresponding polyamide [24,25,26,27]. Due to mild reaction conditions, DSSP is well known to yield high-quality products, as side reactions and thermal degradation are avoided. Moreover, the absence of a melt phase, as well as the relatively simple equipment employed, render the process both industrially appealing and environmentally friendly [28]. Nonetheless, the major disadvantage of DSSP might be its low reaction rate due to the milder operating temperatures.

The DSSP of polyamide monomers or “polyamide salts” has been investigated for the last 50 years for aliphatic and semi-aromatic salts, using various reaction apparatuses [24,25,26,27,28,29,30,31,32,33,34,35,36,37,38,39,40,41,42,43]. During the DSSP of a range of aliphatic polyamide salts (including PA 26, PA 210, PA 46, PA 66, PA 410, PA 610, and PA 126), a well-documented solid-melt transition (SMT) has been observed at reaction temperatures significantly lower than the salts’ melting point [27,29,31,35,38]. Even in recent literature, SMT phenomena have also been observed in the DSSP of PA 55 and PA 512 salts [34,39]. Although the occurrence of SMT is not directly mentioned therein, the authors describe starting from “salt powder” and obtaining a “light, white, plastic product” [39].

Turning to the reaction mechanism, Papaspyrides et al. have suggested that the water formed during DSSP hydrates the polar groups of the hydrophilic polyamide salt, thus leading to the formation of hydrated regions, which in turn exhibit a lower melting point and soon fall into the melt state [28,29,30,31,35,36]. As the reaction proceeds, the molecular weight increases, the hygroscopicity of the system decreases, and the solid state is restored. However, this re-solidification step may cause severe damage to the reactor/stirrer equipment. Thus, the occurrence of SMT is detrimental to the application of DSSP on an industrial scale. Numerous works have attempted to avoid SMT via catalyst incorporation or the selection of an appropriate reactor design [27,30,38,44].

As anticipated, the structure of the polyamide salts significantly influences the outcome of the DSSP reaction and the extent of the occurrence of the SMT phenomenon [26,28,31,35]. Short-chain aliphatic salts (e.g., PA 66) exhibit less organized crystal structures with high amounts of polar ionic bonds, which are hydrophilic and can bind water more easily, thereby providing more sites for SMT. On the contrary, semi-aromatic salts (e.g., PA 4T) exhibit a more rigid and organized network of polar groups; thus, upon DSSP, SMT is totally avoided [37,41]. Similarly, Wolffs et al. employed DSSP to synthesize novel alternating semi-aromatic polyamides with a high biobased content [42].

PA 1212 salt, due to its lower density of polar bonds, could be expected to exhibit some resistance to SMT and allow DSSP to proceed in a true solid state. In this paper, DSSP was initially investigated using a TGA chamber as a micro-reactor. Scaling up from the microscale, the effect of reactor design was also investigated via two laboratory-scale reactors of different designs to discern potential deviations from the aforementioned literature. Detailed morphological observations (via SEM) were combined with several analytical techniques (FTIR-ATR, end-group analysis, dilute solution viscometry) to clarify and understand the reaction mechanism.

## 2. Materials and Methods

### 2.1. Starting Materials

The reactants used for the preparation of polyamide 1212 (PA 1212) were 1,12-diaminododecane (DMDA, Sigma Aldrich^®^, St. Louis, MO, USA) and dodecanedioic acid (DDDA) (Thermo Scientific^TM^, Kandel, Germany). Acetic acid (CH_3_COOH, Chemlab, Zedelgem, Belgium) and benzyl alcohol (BeOH, Penta, Prague, Czech Republic) were used as solvents for the potentiometric titration of polyamide salts. O-cresol (Merck, Hohenbrunn, Germany), phenol (Chemlab), methanol (MeOH, Fischer, Zurich, Switzerland), 1,2-dichlorobenzene (ODCB, Fischer), and lithium chloride (LiCl, Alfa Aesar, Kandel, Germany) were used as solvent systems for potentiometric titration of polyamide samples. Sulfuric acid 99% (Fluka, Seelze, Germany) was used as a solvent for viscosity measurements.

### 2.2. Polyamide Salt Preparation Method

PA 1212 salt was prepared by the solution precipitation technique [28,36]. Two ethanolic solutions of the two reactants were prepared at 40 °C, namely, 19.7 g (98.2 mmol) solid DMDA in 98 mL of ethanol and 21.4 g (93.0 mmol) of DDDA in 93 mL of ethanol. An excess of amine was added to counter any potential amine loss during the dissolution. Then, the acid solution was added dropwise to the amine solution at 0 °C to compensate for the heat generated by the neutralization reaction between DDDA and DMDA. The reaction was performed under reflux to minimize any amine loss as much as possible any amine loss. The salt was instantly precipitated. The resulting suspension was further left to react for 1 h, then filtered and washed with ethanol to remove any excess amine. The sediment was dried under vacuum at 60 °C overnight, then kept for another 24 h under vacuum but also over phosphorus pentoxide. Finally, the salt was received in the form of a very fine white powder.

### 2.3. Direct Solid-State Polymerization (DSSP)

The DSSP of PA 1212 salt was first investigated at the micro scale using a thermal gravimetry analysis chamber (Mettler Toledo TGA/DSC 1 HT, Mettler-Toledo International Inc., Greifensee, Switzerland) as a heating medium and 100 μL aluminum crucibles to simulate SSP laboratory reactor conditions. Ca. 20 mg of dried PA 1212 salt was placed in the crucible, which was then sealed with a lid with a 1.2 mm diameter hole. The samples were inserted into the TGA chamber at 30 °C and heated up to the nominal reaction temperature (*T*_DSSP_) at a rate of 25 °C min^−1^, followed by the final isothermal step at *T*_DSSP_ for periods ranging from 8 to 48 h (t_DSSP_). *T*_DSSP_ was chosen to be ca. 20–30 °C below the melting point of the salt. All experiments were performed under flowing nitrogen at a constant flow rate of 20 mL min^−1^. After the isothermal step, the material was cooled to room temperature. From the data obtained, it is possible to calculate t_1/2_, which is defined as the time at which 50% of conversion is reached. The total mass loss recorded from the TGA experiments is compared to the theoretical loss of water of the PA 1212 salt polycondensation reaction, which can be calculated stoichiometrically (8.4% *w*/*w*) according to Equation (1), at conversion equal to 1. Amine loss is calculated as the deviation of the total mass loss from the theoretical one [32,41].

(1) $$m_{w}=\frac{2\;M_{r,\;H2O}}{M_{r,salt}}$$

Scaling up, DSSP experiments took place in two different reactors, namely Reactors R1 and R2, at 1200 and 600 times upscale, respectively. R1 is a conventional cylindrical autoclave from Parr Instrument Company (Moline, IL, USA) (d = 3 cm, h = 17.5 cm), equipped with a gas inlet and outlet to permit preheated gas to pass through the polymer mass during reaction. R2, also fitted with a gas inlet and outlet, is a fixed-bed reactor with similar dimensions and better at removing the water formed. This reactor has already been presented in a previous work by our group and yielded satisfactory results when applied to the DSSP of PA 612 salt. Within our experimental conditions, a balanced PA 612 prepolymer was obtained while the solid state was maintained throughout the entire process [37,38]. The reactors were loaded with dried PA 1212 salt and purged several times with nitrogen as an inert gas at room temperature. Then, the desired flow rate was set, and heating was started and applied to *T_DSSP_*. After the end of the residence time, the reactor was cooled in an ice bath to 40 °C.

### 2.4. Characterization Methods

#### 2.4.1. Particle Size Distribution

The particle size analysis of PA 1212 salt was determined via laser diffraction in a Laser Malvern Mastersizer Micro 2000 instrument (Malvern Panalytical, Malvern, Worcestershire, UK). Specifically, 500 mg of PA 1212 salt was dispersed in 50 mL of deionized water using an ultrasonic bath and then measured in a Laser Malvern Mastersizer Micro 2000. The dispersity of the sample was calculated using Equation (2):

(2) $$Đ=\;\frac{D(v,0.9)-D(v,\;0.1)}{D(v,\;0.5)}$$

where D(v, 0.9) is the particle size of which 90% of the sample is smaller, D(v, 0.5) is the particle size of which 50% of the sample is smaller, and D(v, 0.1) is the particle size of which 10% of the sample is smaller. Also, the specific surface area is calculated.

#### 2.4.2. Scanning Electron Microscopy (SEM)

SEM images of PA 1212 salt and PA 1212 products were recorded using a Hitachi TM3030Plus Tabletop microscope (Hitachi, Tokyo, Japan), operated at 20 kV. Images were analyzed via ImageJ software (ImageJ 1.53e), using at least 100 measurements to evaluate particle size.

#### 2.4.3. Fourier Transform Infrared Spectroscopy-Attenuated Total Refraction (FTIR-ATR)

FTIR-ATR absorbance spectra of both PA 1212 salt and synthesized polymers were recorded using an Alpha II Bruker Optics Instrument (Bruker Corporation, Billerica, MA, USA). Spectra were received for frequencies from 4000 to 400 cm^−1^ with a resolution of 2 cm^−1^. For each sample, a total of five replicates were measured. Herein, a novel method is proposed for assessing the progress of the reaction. Accordingly, the intensity of two peaks associated with the formation of amide bonds (i.e., at 3307 cm^−1^ and at 1632 cm^−1^), normalized to the intensity at 2918 cm^−1^, is calculated as shown in the following expressions:

$$\frac{I_{3307}}{I_{2918}}\;\mathrm{and}\;\frac{I_{1632}}{I_{2918}}$$

where *I*_3307_ is the intensity of the peak at 3307 cm^−1^ (hydrogen-bonded–NH stretching vibration), *I*_1632_ the intensity of the peak at 1632 cm^−1^ (–C–CO stretching vibration), and *I*_2918_ the intensity of the peak at 2918 cm^−1^ (stretching vibration of the -CH_2_-). In each spectrum, the following ratios are calculated, and the results are presented in a box plot. These ratios express the formation of amide bonds during the polycondensation reaction, normalized to the absorbance of non-reactive methylene units, thereby providing a simple and effective way of indirectly monitoring the reaction progress.

#### 2.4.4. End-Group Concentrations

End-group determination was achieved via potentiometric titration using a Titrando 888 instrument (Metrohm, Herisau, Switzerland). The titrant used for the determination of amine end groups was HClO_4_/MeOH (N ≈ 0.1 meq mL^−1^) and for the carboxyl groups TBAH/BeOH (N ≈ 0.05 meq mL^−1^). For the case of the PA 1212 salt, 0.1% *w/v* solutions of salt in acetic acid for the amine groups and in benzyl alcohol for the carboxyl groups were titrated. For the case of PA 1212 oligomers/polymers, solutions of 0.5% *w/v* in Phenol/MeOH (90/10) for the determination of amine end groups and in o-cresol/ODCB (5%)//LiCl (20% in MeOH) (70/30) were used [36]. The deviation of the mean values was derived through duplicate measurements. The experimental values for the polyamide salts were compared to the theoretical ones as calculated stoichiometrically.

For polyamide samples, it is possible to calculate the number-average molecular weight via Equation (3) [45]:

(3) $$\bar{M_{n}}=\;\frac{2\times 10^{6}}{\left[{-NH}_{2}\right]+[-COOH]}$$

#### 2.4.5. Dilute Solution Viscometry

The intrinsic viscosity (IV) [*η*] of synthesized PA 1212 was measured in sulfuric acid at a concentration of 0.5% *w/v* in a Cannon–Fenske viscometer (K = 0.2308 mm^2^ s^−1^) at 25 °C. Values were obtained by single-point measurement (Equation (4)).

(4) $$[\eta ]=\frac{\sqrt{1+1.5\;\eta_{sp}}-1}{0.75\;C}$$

where C (g mL^−1^) is the concentration and *η*_s*p*_ the specific viscosity of the solution [37]. The deviation of the mean values was derived through duplicate measurements.

#### 2.4.6. Thermal Properties

DSC measurements were performed on a Mettler DSC 1 STAR System. Samples of 8–10 mg were placed in a 40 μL aluminum pan. PA 1212 salt samples followed a single heating cycle from 30 °C to 280 °C with a heating rate of 10 °C min^−1^, while PA 1212 products followed a heating–cooling–heating cycle from 30 °C to 280 °C with a heating and cooling rate of 10 °C min^−1^, under nitrogen flow (25 mL min^−1^).

The TGA analysis was conducted in a Mettler Toledo TGA/DSC 1 HT thermobalance between 30 and 550 °C with a heating rate of 10 °C min^−1^, under nitrogen flow (25 mL min^−1^). The degradation temperature (*T_d_*) was determined at the maximum mass loss rate. For PA 1212 salt samples, the degradation temperature and residue at ca. 200 °C were obtained from data for the first mass loss “step”. The degradation at 5% (*T*_*d*,5%_) was obtained for PA 1212 samples as the temperature at which 5% of the initial mass is lost.

## 3. Results

### 3.1. Polyamide Salt Preparation

#### 3.1.1. Salt Structure and Particle Size

**Figure 1:**
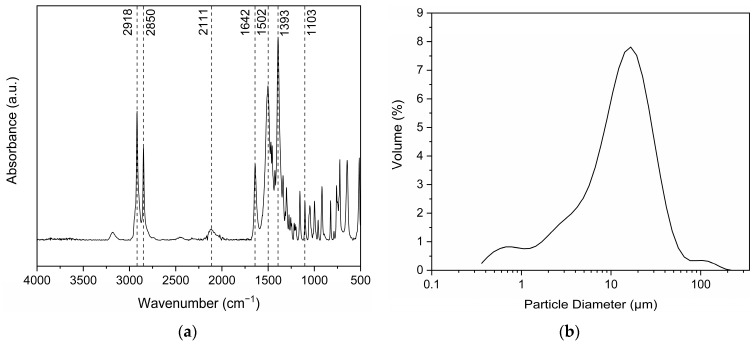
(**a**) FT-IR ATR spectrum of PA 1212 salt; (**b**) particle size diameter as a volume fraction of PA 1212 salt.

Focusing on the verification of the polyamide salt structure, the formation of the ionic bond between the amine and the dicarboxylic acid was confirmed via the ATR-FTIR spectra (Figure 1a) by the peaks at 2111 cm^−1^ corresponding to NH_3_^+^ stretching vibration, those at 1103 cm^−1^ corresponding to NH_3_^+^ transverse rolling vibration, as well as those at 1393 cm^−1^ corresponding to the symmetric stretching vibration of COO^−^. Additionally, the peak at 1642 cm^−1^ corresponds to both the carbonyl bond C=O, but also to the NH_3_^+^ antisymmetric stretching vibration. Furthermore, the two sharp peaks at 2918 cm^−1^ and 2850 cm^−1,^ which correspond to the stretching and bending vibrations of the ethylene bond, indicate the highly aliphatic nature of this salt. Finally, the peak at 1502 cm^−1^ corresponds to the bending vibration of the alkane bond -C-H [36].

**Figure 2:**
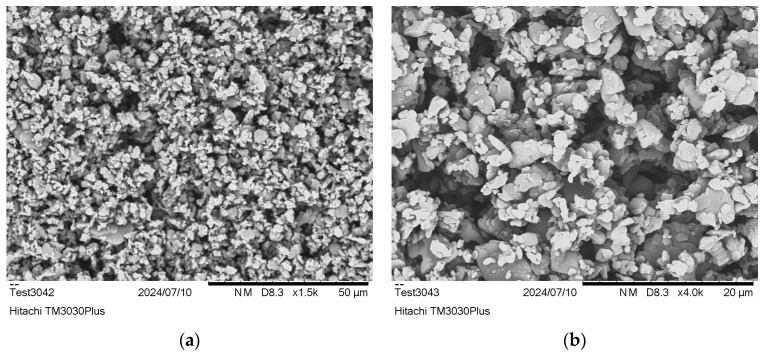
SEM images of PA 1212 salt: (**a**) ×1500 and (**b**) ×4000 magnification.

Dissolution trials in water revealed that the salt remained totally insoluble at room temperature, even in concentrations as low as 0.5%, supporting the assumption that the aliphatic part of the salt predominates over the polar. Therefore, the preparation of aqueous dispersions of the salt was feasible, thus enabling the determination of its particle size distribution and the specific surface area via laser diffraction (Table 1, Figure 1b). Most salt particles are below 30 μm, as indicated by D(v,0.9) (Table 1), which is especially low since for a commercial grade of PA 66 salt (AH Salz^®^ by BASF), less than 2% is below 125 μm. This is to be expected, since the rapid precipitation of the salt during formation prevents the formation of large crystals. In addition, the PA 1212 salt produced has a particularly high specific surface area, which could facilitate polycondensation water removal. The particle size determination of the synthesized PA 1212 salt was found to be in line with SEM observations, which further visualized the formation of small crystals with smooth surfaces and sharp edges (Figure 2).

On the other hand, the results of ImageJ analysis showed that the particle size ranged from 0.8 to 8 μm, with the average being around 2.2 ± 0.2 μm. This is lower than the range determined by laser diffraction and could be attributed to the potential agglomeration of the particles when dispersed during the measurement. Particle size is an important parameter in SSP reactions, as it influences both interior and surface diffusion [46]. In the post-SSP reactions of polyamides and polyesters, for such small particles, interior diffusion is favored, and the rate-determining step is surface diffusion. However, smaller particles are more prone to aggregation. On the other hand, in the case of the DSSP of short-chain aliphatic polyamide salts (e.g., PA 66 salt), the effect of interior diffusion cannot be disregarded due to the salts’ hydrophilic character and hydration, as mentioned in the Papaspyrides model [35]. PA 1212 salt is anticipated to deviate from this model due to the low density of polar groups. Even so, the particles herein are at least 10 times smaller than in the relevant literature [25].

#### 3.1.2. End-Group Analysis and Thermal Properties

End-group analysis proved that the salt produced is well-balanced as both amine and carboxyl groups are similar and close to the theoretical value (4644 meq kg^−1^) (Table 1).

**Figure 3:**
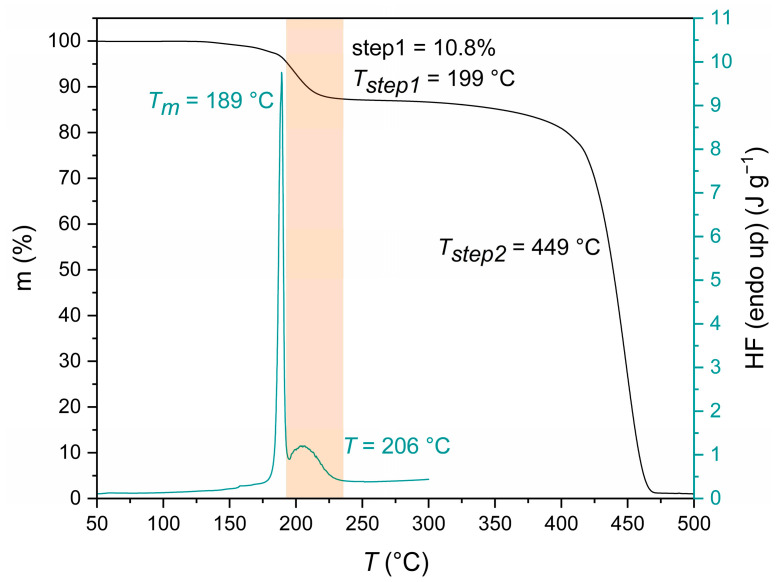
DSC and TGA thermographs of PA 1212 salt.

Turning to the thermal properties of the PA 1212 salt, in the DSC thermographs, two endothermic peaks are apparent (Figure 3), one corresponding to the melting point of the salt (*T_m_* = 190 °C) and a broader one at a higher temperature (206 °C), which corresponds to the evaporation of polycondensation water formed during the melt polymerization of the salt. In addition, unlike the case of other long-chain aliphatic salts (PA 612, 613, 614), no peak related to the accommodation of ethanol was observed, thus emphasizing the non-polar nature of the pertinent salt [36,43]. In the TGA analysis (Figure 3), as in all polyamide salts, two mass loss steps were observed [38]. The first occurred in the range of 185–200 °C with a maximum loss at ca. 200 °C and is attributed to the evaporation of the polycondensation water during polymerization, coinciding with the second DSC peak (as indicated by the colored area in Figure 3). Finally, the second mass loss step (*T_step_*_2_) occurred at approximately 449 °C and corresponds to the degradation of the formed PA 1212.

In conclusion, high aliphatic content, small particle size, and high specific surface area seem to promote the removal of polycondensation water, thus rendering the pertinent salt suitable for DSSP.

### 3.2. DSSP in Microscale

The study of DSSP reactions of polyamide salts at the microscale, using a thermal gravimetry analysis chamber, has been proven to be a valuable and effective tool for studying the kinetics of DSSP reactions, i.e., examining the effect of critical reaction parameters such as temperature, residence time, and nitrogen flow, while using very small quantities and relatively simple equipment. As mentioned in the literature, DSSP reactions of aliphatic salts in general are controlled by diffusion. TGA runs on the microscale promote diffusion, revealing inherent reaction kinetics [24,32,37,42].

**Figure 4:**
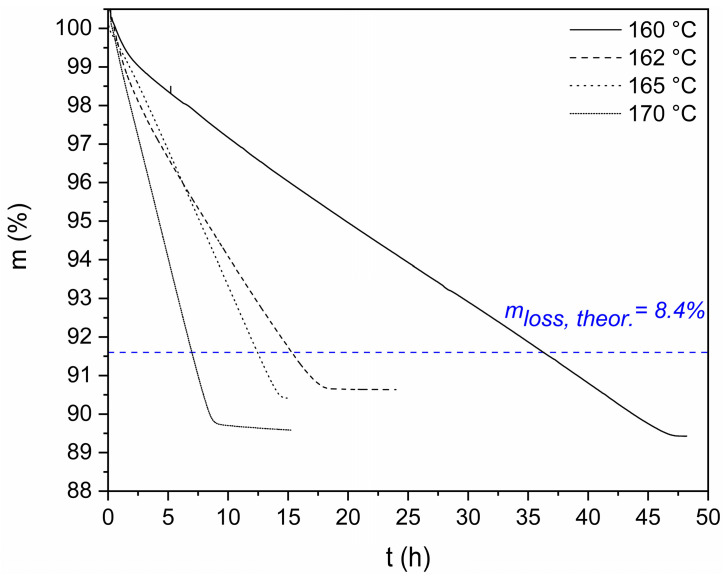
TGA mass loss curves of the DSSP of PA 1212 salt: effect of reaction temperature.

Herein, the effect of reaction temperature was examined (Figure 4, Table 2). The DSSP temperature was fixed at 160 °C, 162 °C, 165 °C, and 170 °C, i.e., ca. 20–30 °C below the melting point of the salt, to limit the appearance of SMT as much as possible. All mass loss curves reached a plateau, indicating that all the theoretical amounts of polycondensation water were removed and full conversion to polyamide was achieved. At the end of the DSSP runs, the morphology of the final product was carefully examined macroscopically. For experiments conducted from 160 to 165 °C, the products were friable between the fingers, revealing a surface softening of the particles.

On the contrary, at the highest reaction temperature (170 °C), reaction products were more sticky but not fully molten, whereas for the aliphatic PA 612 and PA 66 salts, as an example, DSSP products at similar reaction conditions completely fell into the melt state and were received as a bulk mass [27,35,38]. This could be correlated with the fewer polar sites of the PA 1212 salt, resulting in less polycondensation water for hydrating the reacting mass.

The recorded mass loss values exceed the theoretical one (Table 2), since there is an additional volatile diamine loss preceding the polymerization, according to the nucleation and growth mechanism suggested for the DSSP of aliphatic salts [26,28,29,30,31,32,35,38,41]. Specifically, during the nucleation step, the amine component has been found to escape the crystal lattice, thus creating the necessary defects that eventually become the reacting sites that trigger the reaction. Following nucleation, the growth step involves the polycondensation reaction between the amine and carboxyl end groups, releasing water as a byproduct. The reaction proceeds between end groups in proximity [29]. Τhe amine loss is higher at the lower temperature (160°), then decreases to a minimum (165 °C), then increases again at 170 °C. This behavior has also been noticed in our previous work concerning the DSSP of PA 612 [38]. At the lower temperature regime, amine loss is higher due to the low reaction rate, as more amine is swept off until the reaction proceeds at an adequate rate. At a higher temperature regime, although the rate is higher, amine evaporation is also favored, while at intermediate temperatures, there seems to be a balance.

Turning to reaction rate data, it is evident from the very high t_1/2_ values (Figure 4) that the reaction rate is very low, especially at 160 and 162 °C, requiring ca. 48 and 20 h, respectively, to reach full conversion. This very low reaction rate could be attributed to the chemical nature of 1,12-diaminododecane. As mentioned already, the creation of defects in the crystal lattice due to amine sublimation is necessary for the initiation of DSSP. The sublimation of 1,12-diaminododecane is much slower than 1,6-diaminohexane (vapor pressure 0.04 Pa at 25 °C, b.p. 320 °C, compared with vapor pressure 25 Pa at 20 °C and b.p. 204 °C); therefore, defect formation takes longer.

In addition, the effect of reaction temperature on the reaction rate is particularly pronounced, in agreement with previous findings by Volokhina et al. [24,47] and Papaspyrides et al. [28]. In fact, a 2 °C increase from 160 °C to 162 °C results in a 56% decrease in t_1/2_, and a further 3 °C increase (to 165 °C) yields a 24% further decrease in t_1/2_. However, an increase in reaction temperature (from 162 °C to 165 °C), as mentioned above, results in agglomerated but still friable PA 1212 products. Importantly, a complete transition to the melt has never been observed.

It is also worthwhile mentioning that in the previous works of our group on PA 612 and PA 126, under similar reaction conditions, SMT readily occurred with increasing reaction temperature. These findings have been associated with the accumulation of polycondensation water in the reacting mass [28,29,35,38].

### 3.3. DSSP on a Laboratory Scale

#### 3.3.1. Effect of Reactor Design: Comparison of R1 and R2

Based on the aforementioned observations, DSSP was scaled up at the laboratory scale to provide deeper insight into real-life applications. Thus, the runs at the microscale were further compared in the two reactors R1 and R2 (experiments R1_170_24_100 and R2_170_24_100 in Table 3) [38]. The reaction temperature was chosen at 170 °C, as it is the most vigorous reaction condition tested at the microscale. It should be noted here that although the nitrogen flow rate is the same in both experiments (100 mL min^−1^), the calculated gas velocity in R2 is higher (0.20 m min^−1^ compared to 0.14 m min^−1^ in R1), due to the novel design of R2; thus, R2 is considered more efficient in terms of by-product removal.

First, regarding the morphology of the reaction products, in R2, some agglomeration was apparent; however, the product still resembled its initial form, being friable again to free-flowing particles. On the contrary, for R1, the product close to the vessel wall was completely molten. This proves that the retention of solid morphology is achievable only in R2, thus proving the efficiency of this reactor for running DSSP (Figure S1c,d).

It is also worthwhile mentioning that the obtained morphology of PA 1212 from the R1 reactor contradicts our previous work concerning the DSSP of PA 612 salt: under similar experimental conditions, an intense SMT occurred, resulting in a totally molten monolithic PA 612 mass. This observation indirectly proves that PA 1212 behaves very differently from PA 612 (or from other similar hydrophilic aliphatic salts) regarding SMT.

**Figure 5:**
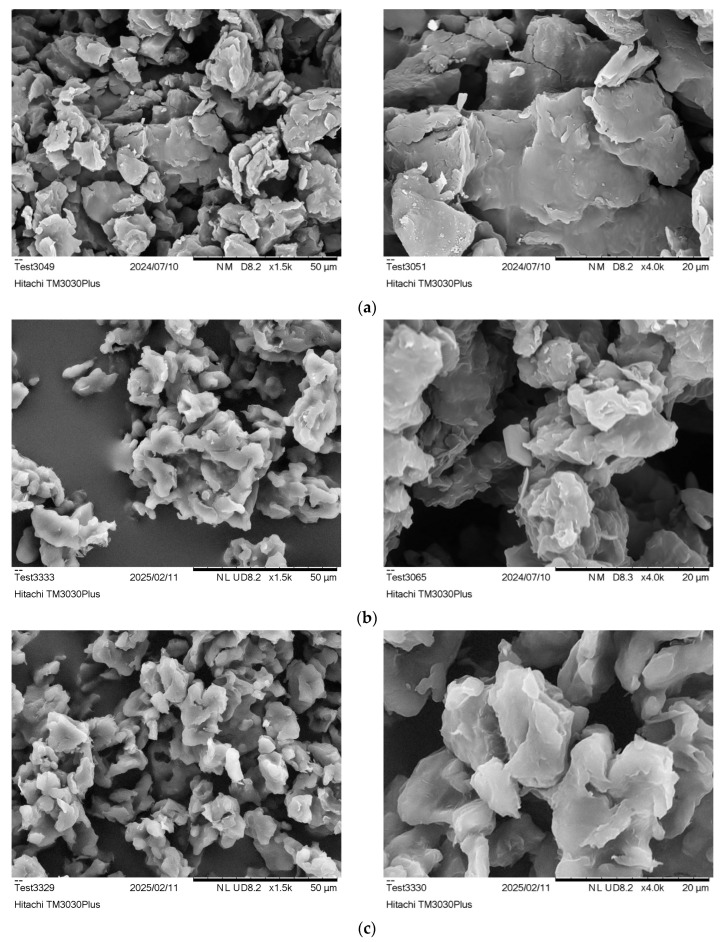

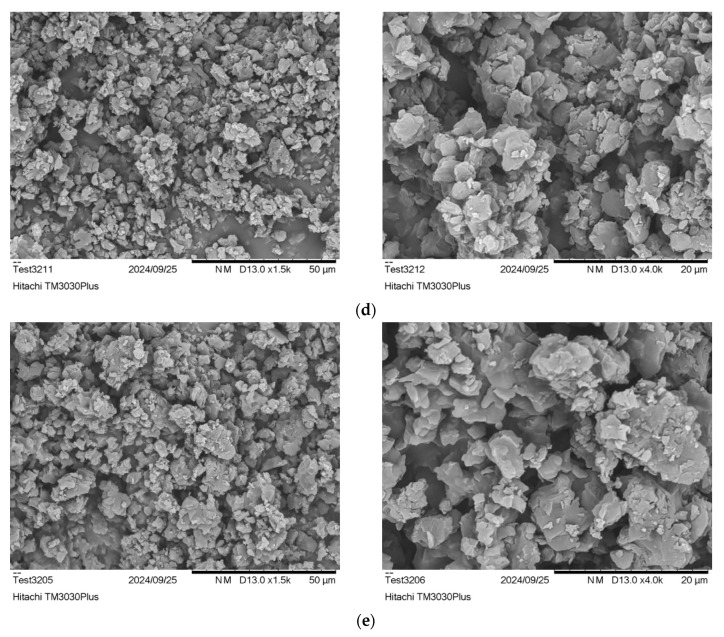
SEM images after DSSP at 170 of (**a**) R1_170_24_100, (**b**) R2_170_24_100, (**c**) R2_170_8_100, (**d**) R2_170_1_100, and (**e**) R2_170_05_100 at 1500× and 4000×.

Macroscopic observations were further verified by the SEM images of the R1_170_24_100 product, where particles with rounded edges and even a few cracks are clearly observed at higher magnification (Figure 5a). Also, the size of these particles is larger than the respective salt particles (Figure 2b), indicating that the salt’s smaller particles have passed through a quasi-melt intermediate to form larger particles. The few observed cracks could be attributed to the escape of polycondensation water and amine during DSSP. On the contrary, SEM images for R2_170_24_100 (Figure 5b) reveal different behaviors, as intense agglomeration is hindered, together with the absence of cracks.

**Figure 6:**
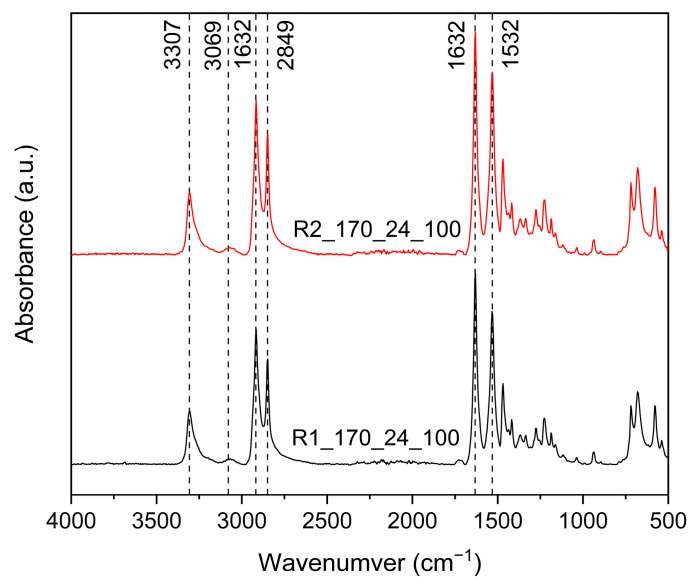
FTIR-ATR spectra of R1_170_24_100 and R2_170_24_100.

The formation of PA 1212 in both reactors was verified via ATR-FTIR spectroscopy (Figure 6). Accordingly, all spectra exhibited absorptions at ca. 3307 cm^−1^ (hydrogen-bonded–NH stretching vibration–Amide Band I), at ca. 3079 cm^−1^ (Amide B overtone of Amide II), at 1632 cm^−1^ (–C–CO stretching vibration–Amide Band I), and at 1532 cm^−1^ (–CN stretching vibration and CONH bend–Amide Band II). Also, the peak at 2111 cm^−1^ corresponding to NH_3_^+^ stretching vibration of the salt is notably absent, thus confirming total transformation to polyamide [36]. Furthermore, the peaks of the R2_170_24_100 spectrum are more pronounced, which could be a qualitative sign of a higher molecular weight.

Indeed, IV measurements (Table 3) verified the latter observation, since R2 exhibited a slightly higher [*η*] value. Nonetheless, the end-group determination (Table 3) resulted in a higher M_n_ for R1. Overall, the differences could be considered negligible, since oligomer formation was practically verified for both reactors.

In terms of diamine loss, a marked increase in the D value was observed compared to the starting PA 1212 salt (D > 300 meq kg^−1^ compared to D = 80 meq kg^−1^) (Table 3). This indicates significant amine loss, despite the relatively high boiling point of 1,12-diaminododecane, as mentioned in Section 3.2. This is attributed to the very high reaction time (24 h). Although most of the diamine is lost in the beginning and is necessary for the creation of defects in the crystal lattice of the salt, a significant percentage is also lost during the entire course of the reaction. Amine loss was higher in R2 due to the higher inert gas velocity, which, apart from polycondensation water, also forces the amine to sweep off, thus explaining the slightly decreased [-NH_2_] and M_n_ values of the R2_170_24_100 sample.

**Figure 7:**
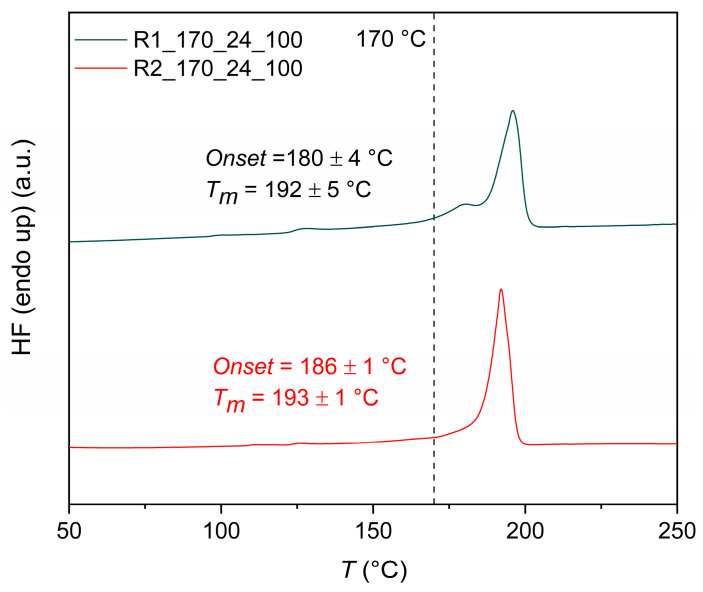
DSC of R1_170_24_10 and R2_170_24_100 products.

Turning to the DSC, the curves of R1_170_24_100 and R2_170_24_100 products present an endotherm peak at 192–193 °C during the 1st heating step, corresponding to the melting of PA 1212 products (Figure 7). It is interesting to note that the melting point of the reacting mass does not increase with increasing conversion after DSSP, so practically the melting points of the 1212 salt and of the PA1212 formed almost overlap. This is because the amide bonds formed are few and interrupted by large aliphatic chains. In other words, there are few polar groups between which hydrogen bonds can be formed.

#### 3.3.2. Effect of Reaction Temperature

The TGA experiments at the microscale were upscaled in the R2 reactor at 160–170 °C, while maintaining a residence time of 24 h and a nitrogen flow rate of 100 mL min^−1^ (R2_160_24_100, R2_162_24_100, R2_165_24_100, and R2_170_24_100) (Table 3).

As to their morphology, all products showed the same behavior as that observed in the microscale experiments, i.e., they were easily removed from the reactor and turned into powder with gentle manual stress.

**Figure 8:**
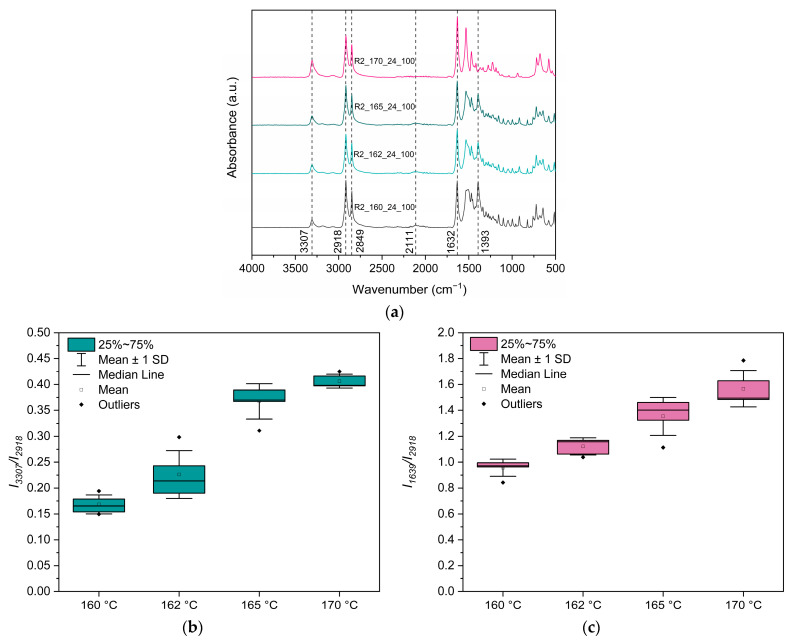
(**a**) ATR-FTIR spectra of PA 1212: Effect of reaction temperature. Evolution of the (**b**) *I*_3307_*/I*_2918_ ratio and (**c**) *I*_1639_*/I*_2918_ ratio with increasing reaction temperature.

Turning first to the structure of PA 1212 products, some polyamide formation was verified by ATR-FTIR data in R2_160_24_100, R2_162_24_100, and R2_165_24_100 experiments, as the intensity of the peak at 2111 cm^−1^ was found to be significantly decreased (Figure 8a). Furthermore, the peak at 1393 cm^−1^ is apparent, indicating some residual PA 1212 salt. This peak intensity decreases with increasing reaction temperature and disappears completely from the spectrum only in the case of R2_170_24_100, indicating that the salt has fully reacted to polyamide, further verified by the complete absence of the 2111 cm^−1^ peak. Herein, a novel FTIR-ATR-based method is introduced, aimed at monitoring the progress of polyamidation by calculating the ratios *I*_3307_*/I*_2918_ and *I*_1639_*/I*_2918_. This method was applied to all the samples in this work. Both ratios increase with increasing reaction temperature, indicating the formation of amide bonds and increasing molecular weight, as expected (Figure 8b,c).

In agreement with FT-IR ATR data, the high end-group content verified the presence of salt in the R2_160_24_100 and R2_162_24_100 products (Table 3). Therefore, at 160 °C and 162 °C, the reaction does not reach an acceptable conversion. At 165 °C, the sample still contains some residual salt (as verified by FTIR), but it comprises mostly oligomers with a low [*η*]. At 170 °C, polyamide formation is confirmed by FT-IR ATR, by the end-group content, and the highest achieved [*η*] value of 0.50 dL g^−1^ (Table 3). At this point, it must be stated that the very low Mn values reported in Table 3 (300–400 g mol^−1^) reflect the low reaction conversion attained, revealing a substantial fraction of unreacted salt contained in the final product.

It is also worth noting that the amine loss (as represented by the D values in Table 3) follows the same trend as in microscale experiments. Specifically, D values are very high at 160 °C (585 meq kg^−1^), reaching a minimum at 165 °C (193 meq kg^−1^), then increasing slightly again at 170 °C (470 meq kg^−1^). This confirms the mechanism presented in Section 3.2, linking amine loss to reaction rate.

**Figure 9:**
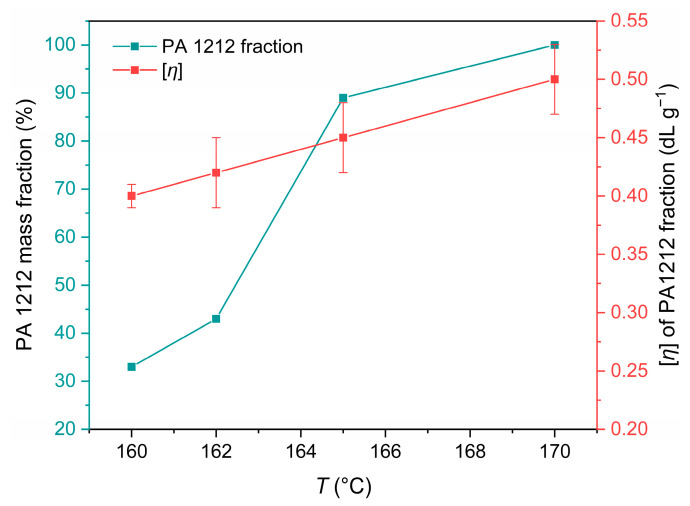
Pure PA 1212 mass fraction and intrinsic viscosity in DSSP products: R2_160_24_100, R2_162_24_100, R2_165_24_100, and R2_170_24_100.

In order to get more information about the reaction conversion at the lower temperatures, products from 160 °C, 162 °C, and 165 °C (R2_160_24_100, R2_162_24_100, and R2_165_2_100) were washed with acetic acid to remove salt residues and then filtered and washed with water to isolate the converted polyamide fraction. After drying, the mass of the polyamide was weighed, and the percentage of PA 1212 in each sample was determined. The PA 1212 yield for experiments containing unconverted salt was in the range of ca. 30–90%, according to Figure 9. In these “clean” PA 1212 sediments, the IV was also measured (Figure 9). As expected, with increasing reaction temperature, the conversion to PA 1212 also slightly increases. However, the range of the [*η*] values of the “clean” PA 1212 is very limited (from 0.40 to 0.50 dL g^−1^). Correlating these values to the morphology of PA 1212 products, it is evident that at 160 °C and 162 °C, the absence of agglomeration is linked to the low conversion and high salt content of R2_160_24_100 and R2_162_24_100.

Considering the aforementioned data, the effect of temperature is particularly pronounced, as in microscale experiments. Increasing the reaction temperature from 160 °C to 162 °C leads to a 30% increase in polymer content. A further 3 °C increase from 162 °C to 165 °C leads to a 103% increase in the polymer content and the formation of oligo-amides. At 165 °C, there seems to be a threshold above which the reaction proceeds at a higher rate. The [*η*] value increases even more when the reaction temperature is raised to 170 °C. It is therefore confirmed that aliphatic polyamide salts have a “high temperature coefficient,” as discussed also in Section 3.2. and previous literature [24,26,31].

Based on the experiments at the microscale, it is well proven that for all temperatures (except 160 °C), the reaction reaches a plateau after 24 h (Figure 4). Meanwhile, at the laboratory scale, full conversion was achieved only at 170 °C, with all other products containing residual salt. In fact, in TGA, there are optimal mass and heat transfer conditions, but when scaling up to the laboratory level, the limiting effect of amine and byproduct diffusion becomes pronounced, as expected.

#### 3.3.3. Assessment of the Reaction Mechanism

For a more comprehensive investigation of the reaction mechanism, shorter time DSSP runs of 30 min (R2_170_05_100), 1 h (R2_170_1_100), 2 h (R2_170_2_100), 6 h (R2_170_6_100), and 8 h (R2_170_8_100) were performed in R2 (Table 3). The reaction temperature was kept at 170 °C, as it was proven to ensure complete conversion to polyamide after a residence time of 24 h.

**Figure 10:**
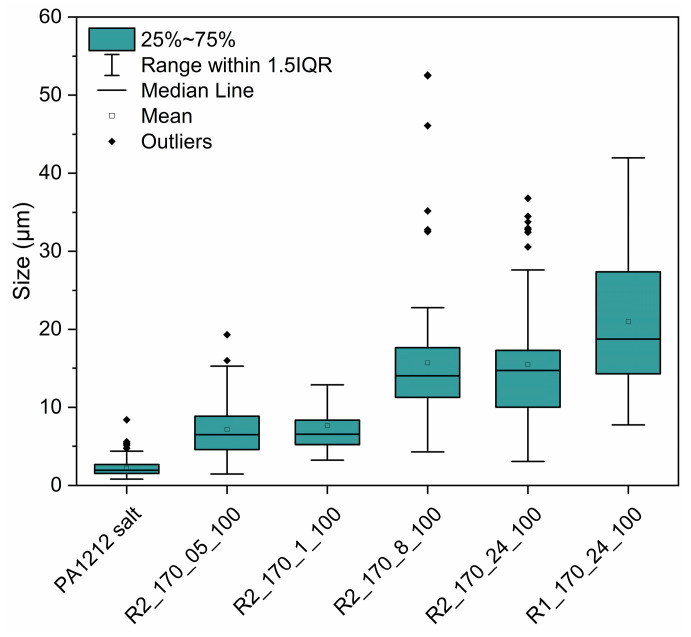
Size analysis of DSSP products from SEM images.

Macroscopically, up to 2 h of reaction time, no significant morphological changes were observed, while for 6, 8, and 24 h, friable products were obtained. Nonetheless, when examining the SEM images (Figure 5c–e), morphological changes were apparent as early as 30 min of residence time, although the conversion was still very low (Figure 5e). In fact, as the reaction time increased further, smaller crystals clearly passed through a quasi-melt state and agglomerated to larger particles, e.g., after 8 h (Figure 5c). The latter is also shown in Figure 10, where the narrow particle size distribution (2–7 μm) of the initial PA 1212 salt gradually shifts towards higher values with increasing reaction time.

**Figure 11:**
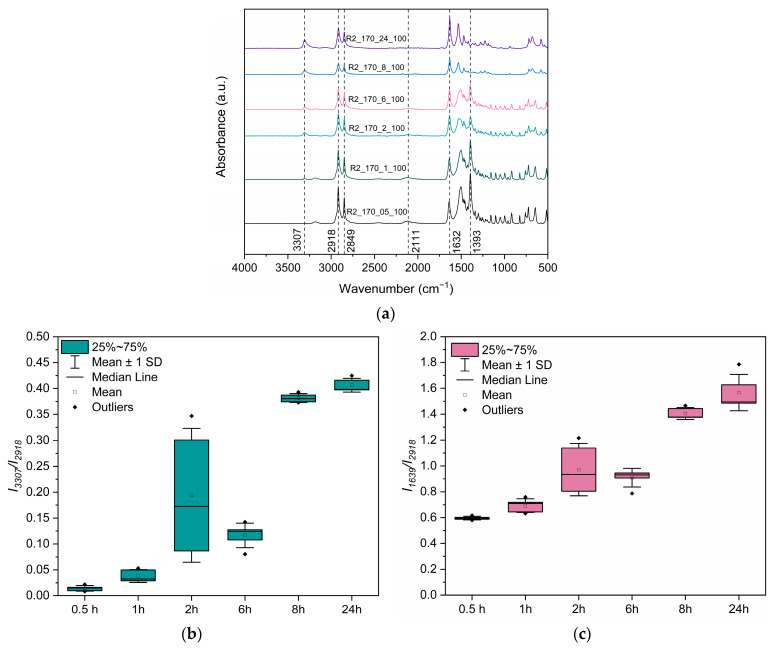
(**a**) FTIR-ATR spectra of R2_170_05_100, R2_170_1_100, R2_170_2_100, R2_170_6_100, R2_170_8_100, and R2_170_24_100. Evolution of the (**b**) *I*_3307_*/I*_2918_ and (**c**) *I*_1632_*/I*_2918_ ratios with increasing reaction time.

Examining the ATR-FTIR data (Figure 11), the samples at 0.5 h, 1 h, 2 h, and 6 h present the peak at 1393 cm^−1^, indicating the presence of salt (Figure 11a). Samples at 2 h and 6 h also present the peak at 3307 cm^−1^, verifying the formation of amide bonds and the synthesis of some oligomers. Spectra of samples from 8 h and 24 h do not present any peaks associated with salt presence, indicating that the salt was fully converted. Moreover, both *I*_3307_*/I*_2918_ and *I*_1639_*/I*_2918_ ratios increase with reaction time, which could be associated with the formation of amide bonds in the sample (Figure 11b,c). Between 6 and 8 h, a sharp increase in both ratios is detected, which may be related to a sudden increase in molecular weight during this period, in harmony with Figure 5c and Figure 10. Furthermore, the distribution is narrower at very low conversions (0.5 and 1 h) and at higher conversions (8 and 24 h), whereas it is broader at intermediate times (2 h). This occurs because at intermediate stages, both salt and polymer are likely to coexist, and sampling may capture regions containing either one or the other, or a mixture of both.

**Figure 12:**
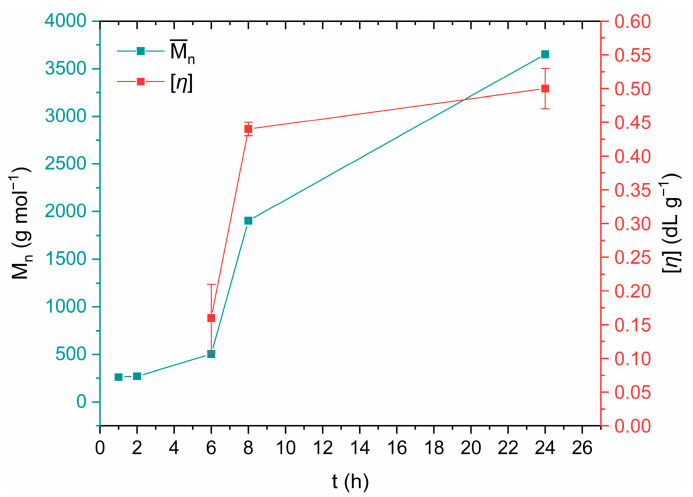
Evolution of the number-average molecular weight and the intrinsic viscosity of DSSP products with increasing reaction time.

To validate the efficiency of the aforementioned FT-IR ATR method, DSSP products were also evaluated via end-group analysis and intrinsic viscosity measurements (Table 3). Due to the very low degree of conversion, IV measurements were not feasible for samples with reaction times shorter than 6 h. Up to 2 h, the reaction proceeds relatively slowly, while the most significant increase in molecular weight occurs between 6 and 8 h (an increase of 277% in ${\bar{M}}_{n}$, and of 175% in [*η*]) (Figure 12). This observation confirms the ATR-FTIR results, while during this time interval, the most pronounced morphological deterioration is also observed (Figure 5c and Figure 10). From 8 to 24 h, ${\bar{M}}_{n}$ increases by 92% and [*η*] by 14%. It is also observed that the parameter D, which expresses the deviation from the stoichiometric equivalence of end groups, increases over time due to amine loss during DSSP (Table 3).

Turning further to mechanism aspects, Papaspyrides et al. suggested, a long time ago, that the DSSP of typical aliphatic polyamide salts follows three steps in sequence: (i) an initial induction step occurring in the solid state and marked by low conversion levels where practically the defects necessary to initiate polycondensation are created in the crystal structure of the salt accompanied by amine loss; (ii) the SMT step, during which the reacting mass falls to the melt state due to hydration of the polar groups, leading to a rapid increase in conversion, while following second- or third-order kinetics, as suggested by Flory, and depending on the absence or presence of catalysts, respectively [45]; and finally, (iii) a re-solidification step, in which, due to the increases in melting point and [*η*], the reacting mass reverts to the solid state [28]. Herein, this mechanism seems credible, as the following steps can be distinguished: (i) an induction step and (ii) an intermediate step of agglomeration, but without any full transition to the melt state, however, contributing to a rapid increase of the conversion (Figure 12). The re-solidification step (iii) is not distinguishable since the reacting mass never loses its solid character. This is because the melting points of both the salt and the polyamide overlap (i.e., ca. 190 °C), as proved by DSC analysis in Figure 3 and Figure 7. Last but not least, the curve presented in Figure 12 resembles a typical nucleation and growth model.

In conclusion, the DSSP of PA1212 salt can efficiently run without passing through a melt state (passing only through a surface softening or a mild agglomeration stage). Moreover, the absence of the re-solidification step may appeal to larger-scale and industrial applications, since the reacting mass remains “friable” at all times.

## 4. Conclusions

The present work focuses on the study of the DSSP of PA 1212 salt. The reaction was initially studied at the microscale and then scaled up in two different laboratory-scale reactors. The effect of critical parameters, such as residence time and reaction temperature, was examined, aiming to investigate the reaction mechanism and compare it with previous literature data on the DSSP of aliphatic polyamide salts. The progress of the DSSP runs was monitored via both molecular weight evolution and morphological observations. The former was also indirectly monitored by a novel method of FTIR-ATR analysis. Notably, key deviations from the well-established SMT mechanism were observed. The DSSP induction step was proven to last for a short time interval, whereas the melt transition step was replaced by a mild agglomeration step due to the hydrophobic nature of the PA 1212 salt. Thus, the melting point of the reacting mass does not increase with increasing molecular weight, thereby eliminating the industrially undesirable re-solidification step. The latter comprises an important aspect, as it opens a new pathway for potential industry-viable applications of DSSP, and consequently, more sustainable polyamide production.

## Figures and Tables

**Table 1:** PA 1212 salt properties (end-group analysis, particle size distribution, and thermal properties).

**End-Group Analysis**											
**[NH_3_^+^]_theor_ = [COO^−^]_theοr_ (meq kg^−1^)**			**[NH_3_^+^] (meq kg^−1^)**			**[COO^–^] (meq kg^−1^)**			**D = [COO^–^] − [NH_3_^+^] (meq kg^−1^)**		
4644			4562 ± 37			4642 ± 99			80		
**Particle Size Distribution via Laser Diffraction**											
**D(4,3) (μm)**	**D(v, 0.1) (μm)**			**D(v, 0.5) (μm)**		**D(v, 0.9) (μm)**		**Đ**			**Specific surface area (m^2^/g)**
15.65 ± 0.03	2.20 ± 0.07			11.92 ± 0.36		29.90 ± 0.69		2.32 ± 0.01			1.30 ± 0.01
**Thermal Properties**											
***T_m_*** ** (°C)**		**Δ*H_m_* (J g^−1^)**			***T*_step1_ (°C)**		***T*_step2_ (°C)**			**Step1 (%)**	
190 ± 2		167 ± 12			200 ± 1		450 ± 2			11.0 ± 0.2	

**Table 2:** Kinetic data of the DSSP of PA 1212 salt: effect of reaction temperature.

*Τ*_DSSP_ (°C)	N_2_ (mL min^−1^)	t_1/2_ (h)	Mass Loss (%)	Amine Loss (%)
160	25	21.2	10.6	2.2
162	25	9.2	10.3	1.9
165	25	7.0	10.1	1.7
170	25	4.4	10.5	2.1

**Table 3:** Experimental conditions, end-group concentration, and intrinsic viscosity for PA 1212 products.

Experiments Reactor_T_t_flow Rate	*T*_DSSP_ (°C)	t_DSSP_ (h)	Volumetric Flow Rate (mL min^−1^)	Gas Velocity (m min^−1^) ^[1]^	[-NH_2_] (meq kg^−1^)	[-COOH] (meq kg^−1^)	$\bar{M_{n}}$ (g mol^−1^)	D = [COOH] − [NH_2_] (meg kg^−1^)	[*η*] (dL g^−1^)
Runs at 170 °C: Effect of reactor design (Comparison of R1 and R2)									
R1_170_24_100	170	24	100	0.16	46 ± 8	395 ± 21	4500	349	0.41 ± 0.02
R2_170_24_100	170	24	100	0.20	39 ± 1	509 ± 50	3700	470	0.50 ± 0.03
Effect of reaction temperature in Reactor R2									
R2_160_24_100	160	24	100	0.20	2685 ± 101	3271 ± 152	300	585	0.40 ± 0.01 ^[2]^
R2_162_24_100	162	24	100	0.20	2135 ± 37	2439 ± 40	400	304	0.42 ± 0.03 ^[2]^
R2_165_24_100	165	24	100	0.20	524 ± 20	717 ± 35	1500	193	0.45 ± 0.03 ^[2]^
R2_170_24_100	170	24	100	0.20	39 ± 1	509 ± 50	3700	470	0.50 ± 0.03
Effect of residence time in reactor R2 at 170 °C									
R2_170_05_100	170	0.5	100	0.20	n.d. ^[3]^	n.d. ^[3]^	n.d. ^[3]^	n.d. ^[3]^	n.d. ^[3]^
R2_170_1_100	170	1	100	0.20	3701 ± 24	3936 ± 155	260	235	n.d. ^[3]^
R2_170_2_100	170	2	100	0.20	3142 ± 145	3595 ± 150	270	383	n.d. ^[3]^
R2_170_6_100	170	6	100	0.20	1718 ± 36	2032 ± 101	500	314	0.16 ± 0.05
R2_170_8_100	170	8	100	0.20	150 ± 13	575 ± 7	1900	425	0.44 ± 0.01
R2_170_24_100	170	24	100	0.20	39 ± 1	509 ± 50	3700	470	0.50 ± 0.03

^[1]^ Nitrogen velocity is calculated via the volumetric flow rate and the reactor cross-section variables. ^[2]^ Determined for the converted part. ^[3]^ n.d.: not determined.

## Data Availability

The original contributions presented in this study are included in the article/Supplementary Material. Further inquiries can be directed to the corresponding author.

## Supplementary Materials

The following supporting information can be downloaded at https://www.mdpi.com/article/10.3390/polym18010101/s1: Figure S1: Indicative photos of samples: (a) TGA_160_24_100, (b) TGA_170_24_100, (c) R1_170_24_100, and (d) R2_170_24_100.

## Abbreviations

The following abbreviations are used in this manuscript:

DSSP	Direct solid-state polymerization
SMT	Solid melt transition
DDDA	1,12-Dodecanedioic acid
DMDA	1,12-Diaminododecane
