# Supplementary figures and images for: Direct Solid-State Polymerization of Highly Aliphatic PA 1212 Salt: Critical Parameters and Reaction Mechanism Investigation Under Different Reactor Designs
Source: Polymers (Basel). 2025 Dec 29;18(1):101. doi: 10.3390/polym18010101 (PMC12787641; doi:10.3390/polym18010101)

## Supplementary Information

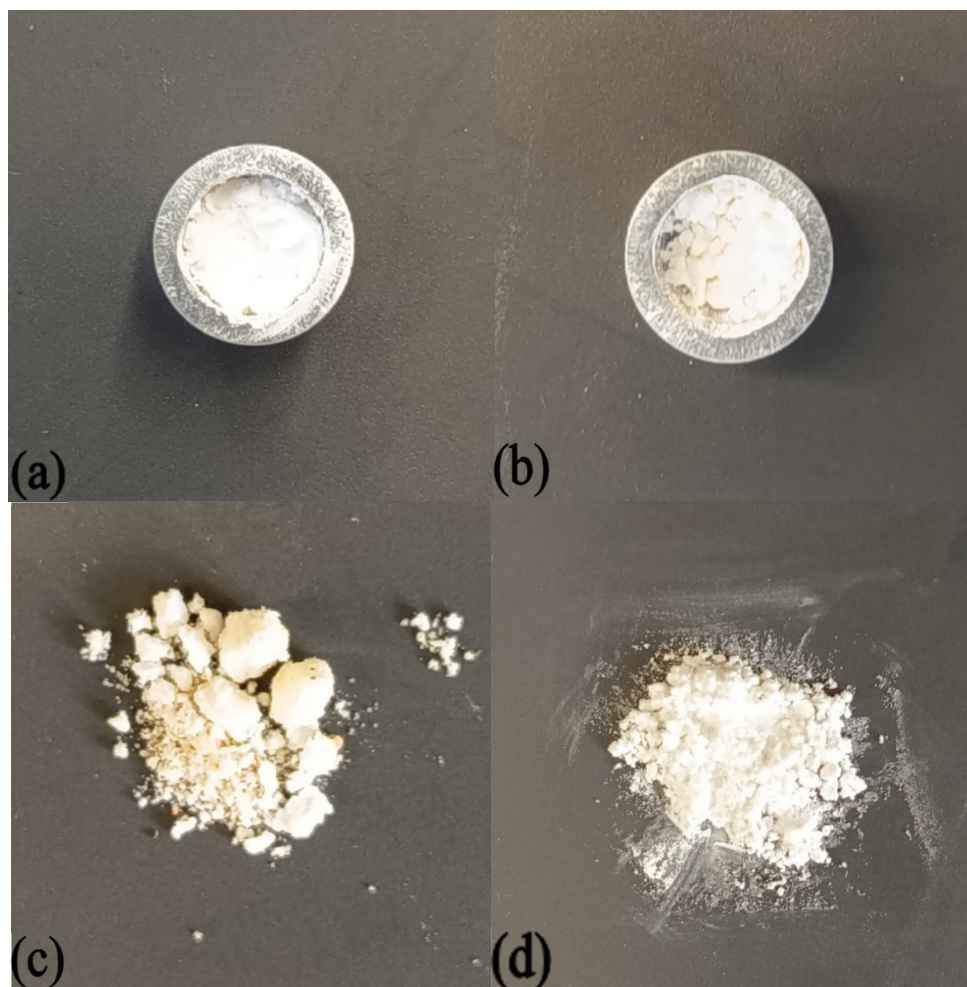

Figure S1: Indicative photos of samples: (a) TGA\_160\_24\_100, (b) TGA\_170\_24\_100, (c) R1\_170\_24\_100 and (d) R2\_170\_24\_100

Supplement: Supplementary file 1 [file polymers-18-00101-s001.zip › polymers-4044581-supplementary.pdf]
